# A laparoscopic one anastomosis gastric bypass with wrapping versus nonwrapping fundus of the excluded part of the stomach to treat obese patients (FundoRingOAGB trial): study protocol for a randomized controlled trial

**DOI:** 10.1186/s13063-022-06252-6

**Published:** 2022-04-07

**Authors:** Oral Ospanov, Galymzhan Yeleuov, Alexandr Fursov, Bakhtiyar Yelembayev, Roman Fursov, Zhenis Sergazin, Adil Mustafin

**Affiliations:** Department of Surgical Disease and Bariatric Surgery, Astana Medical University, Nur-Sultan, Kazakhstan

**Keywords:** Morbid obesity, Gastroesophageal reflux disease, Minigastric bypass, One anastomosis gastric bypass, Fundoplication, FundoRingMGB, FundoRingOAGB

## Abstract

**Background:**

Laparoscopic one anastomosis gastric bypass (LOAGB) is a simple variation of gastric bypass and has gained worldwide popularity with clinical outcomes similar to laparoscopic Roux-en-Y gastric bypass (LRYGB) for weight loss and management of comorbidities. However, biliary reflux to the esophagus negates the benefits of LOAGB. In addition, weight gain after LOAGB and after LRYGB is a major problem in bariatric surgery.

The aim of this article is to describe the design and protocol of a randomized controlled trial comparing the outcomes of two methods of LOAGB: experimental method with wrapping versus standard method nonwrapping fundus of the excluded part of the stomach to prevent weight regain and biliary reflux after LOAGB.

**Methods:**

The study was designed as a single-center prospective, interventional, randomized controlled trial. Masking: None (open label). Allocation: randomized. Enrollment: 100 obese patients. The relevant ethics committee approved the trial protocol.

The endpoints (body mass index, bile reflux in the esophagus, other reflux symptoms) will be assessed presurgery and postsurgery (12, 24, and 36 months postoperatively).

**Discussion:**

With its 3-year follow-up time, this RCT will provide important data on the impact of wrapping the fundus of the excluded part of the stomach to prevent weight regain and biliary reflux after LOAGB.

**Trial registration:**

ClinicalTrials.govNCT04834635. Registered on 8 April 2021.

## Administrative information

Data of FundoRingOAGB trial.

**Table:** 

Title {1}	A laparoscopic one anastomosis gastric bypass with wrapping versus nonwrapping fundus of the excluded part of the stomach to treat obese patients (FundoRingOAGB trial): study protocol for a randomized controlled trial
Trial registration {2a and 2b}.	ClinicalTrials.gov, NCT04834635
Protocol version {3}	Registered on 8 April 2021. Version 8 April 2021.
Funding {4}	The work will be supported by The Society of Bariatric and Metabolic Surgeons of Kazakhstan, Nur-Sultan, Kazakhstan, grant number 2021/1.
Author details {5a}	Oral Ospanov1 Galymzhan Yeleuov1, Alexandr Fursov1, Bakhtiyar Yelembayev1, Roman Fursov1, Zhenis Sergazin1, Adil Mustafin1 1. Department of Surgical Disease and Bariatric Surgery, Astana Medical University, Nur-Sultan, Kazakhstan
Name and contact information for the trial sponsor {5b}	The Society of Bariatric and Metabolic Surgeons of Kazakhstan, Nur-Sultan, Kazakhstan
Role of sponsor {5c}	The funder had no role in the study design, data collection, data analysis, data interpretation, or writing of the manuscript

## Introduction

### Background and rationale {6a}

Typical early weight loss following bariatric surgery ranges from 47 to 80% of excess weight [1]. However, typical weight regain is 15–25% of that lost weight [2]. Weight regain after surgery is a major problem in bariatric practice. Therefore, adjustable bands and rings are used, such as “FobiRing” for banded gastric bypass [3]. However, foreign material can cause complications, such as erosion of the stomach wall [4]. Therefore, surgeons avoid the use of mechanical devices for banded gastric bypass.

Currently, laparoscopic one anastomosis gastric bypass (LOAGB) or minigastric bypass (MGB) is a common bariatric procedure for treating obesity [5]. The greatest criticism of the LOAGB is for the likelihood of biliary reflux [6]. Biliary reflux resistant to medical treatment has an incidence of 0.6–10% after one anastomosis gastric bypass and may be a reason for revisional surgery [7].

In addition, along with obesity, gastroesophageal reflux disease (GERD) is steadily increasing worldwide, and antireflux surgery must be performed simultaneously with bariatric surgery in obese patients [8]. In these cases, fundoplication may be performed using the fundus of the excluded part of the stomach [9]. Colpaert J et al. reported good short-term results with modified Nissen fundoplication for patients with reflux after RYGB [10]. They opted to perform the same procedure to slow down the passage of food through the gastric pouch. This procedure can be performed as a revision procedure after Roux-en-Y gastric bypass for the treatment of postoperative dumping syndrome [11].

In the literature, we did not find data on the use of a similar method for primary procedures and examples of use for patients without GERD to prevent biliary reflux in one anastomosis gastric bypass and de novo postoperative reflux esophagitis.

### Objectives {7}

We hypothesize that total fundoplication can not only treat GERD but also significantly prevent the return of weight, such as after banded gastric bypass, prevent postoperative bile reflux in the esophagus, and decrease de novo GERD.

The aim of this article is to describe the design and protocol of a randomized controlled trial comparing the outcomes of two methods of LOAGB: the experimental method with wrapping versus the standard method nonwrapping fundus of the excluded part of the stomach.

The main aim of the study are as follows:

1. Comparing the primary outcome measure: change in body mass index in both groups and weight regain.
2. Comparing the secondary outcome measures: postoperative bile reflux in the esophagus; assessment of GERD if preoperative GERD symptoms (GERD-HRQL) were present or if GERD symptoms occurred postoperatively (de Novo GERD).

### Trial design {8}

The study was designed as a single-center prospective, interventional, randomized controlled trial. Masking: None (Open Label) Allocation: randomized. Enrollment: 100 obese patients.

## Methods: participants, interventions, and outcomes

### Study setting {9}

Participant data will be collected from “Green Clinic,” Nur-Sultan, Kazakhstan. All operations are performed by one surgeon.

### Eligibility criteria {10}

Inclusion criteria:

- Obese patients had a body mass index (BMI) from 30 to 50 kg/m^2^.
- The patient is generally fit for anesthesia (ASA grading 1–2) and surgery.
- The patient commits to the need for long-term follow-up.

Exclusion criteria:

- Patients with superobesity—BMI more than 50 kg/m^2^.
- Prosthetic (mesh) Hiatal herniorrhaphy or large hiatal hernia
- Esophageal shortening
- Los Angeles Classification of Esophagitis (LA grade) C or D reflux esophagitis
- History of surgery on the stomach or esophagus
- Less than 18 or more than 60 years of age
- Not fit for bariatric surgery
- Psychiatric illness
- Patients unwilling or unable to provide informed consent

The drop out criteria:

1. Refusal to participate
2. Patient not available for long-term follow-up

### Who will take informed consent? {26a}

Written informed consent will be obtained from all participants or their authorized representatives.

### Additional consent provisions for collection and use of participant data and biological specimens {26b}

Not applicable.

## Interventions

### Explanation for the choice of comparators {6b}

The choice of a comparator—the method of laparoscopic one-anastomosis (mini) gastric bypass by M. Carbajo-based long-term follow-up results in 1200 patients [12]

### Intervention description {11a}

**Fig. 1:**
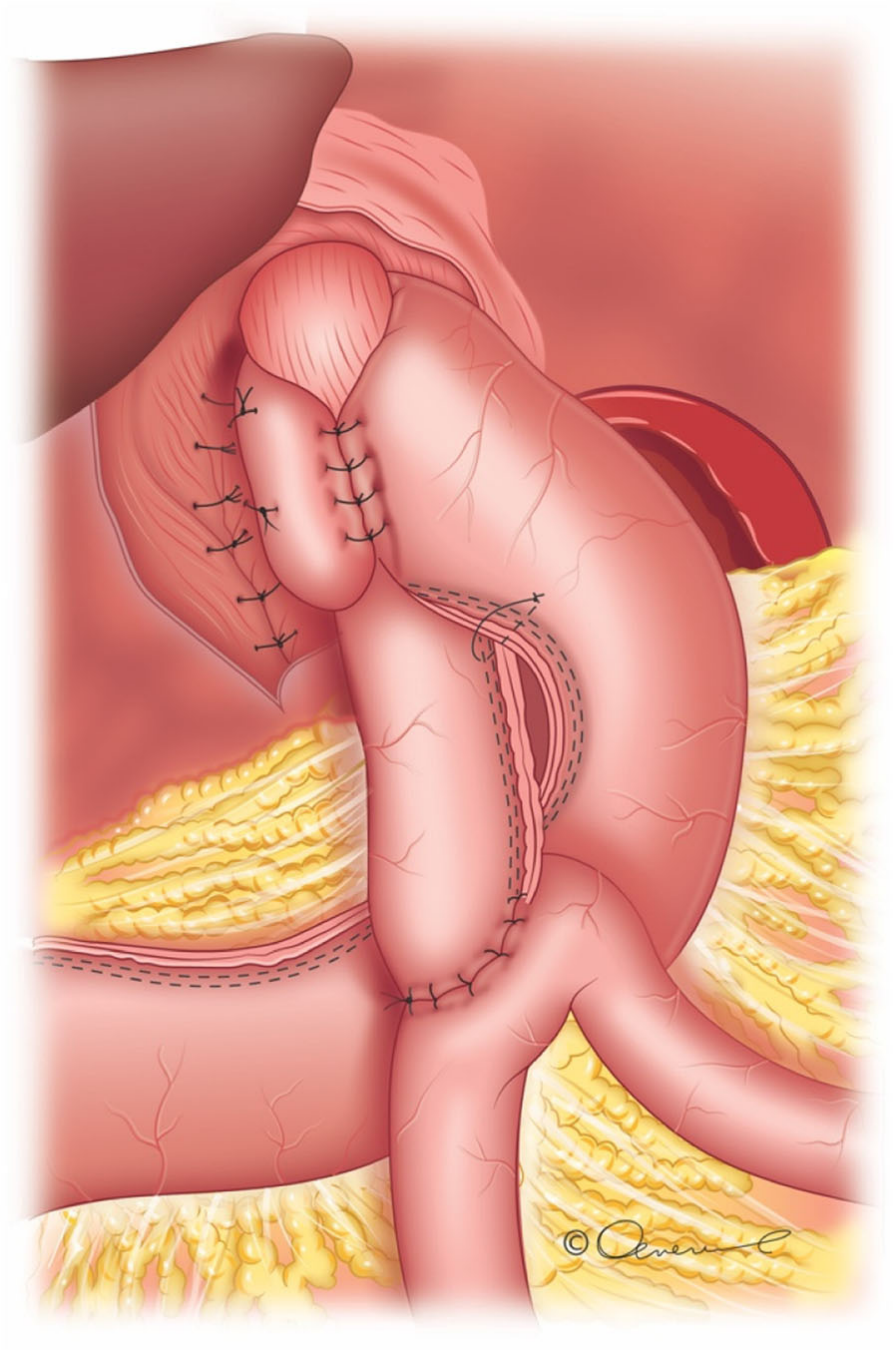
FundoRing OA(M)GB - Laparoscopic anastomosis (mini) gastric bypass with total wrapping of the fundus of the gastric excluded part and suture cruroplasty.© 2021 Oral Ospanov

**Fig. 2:**
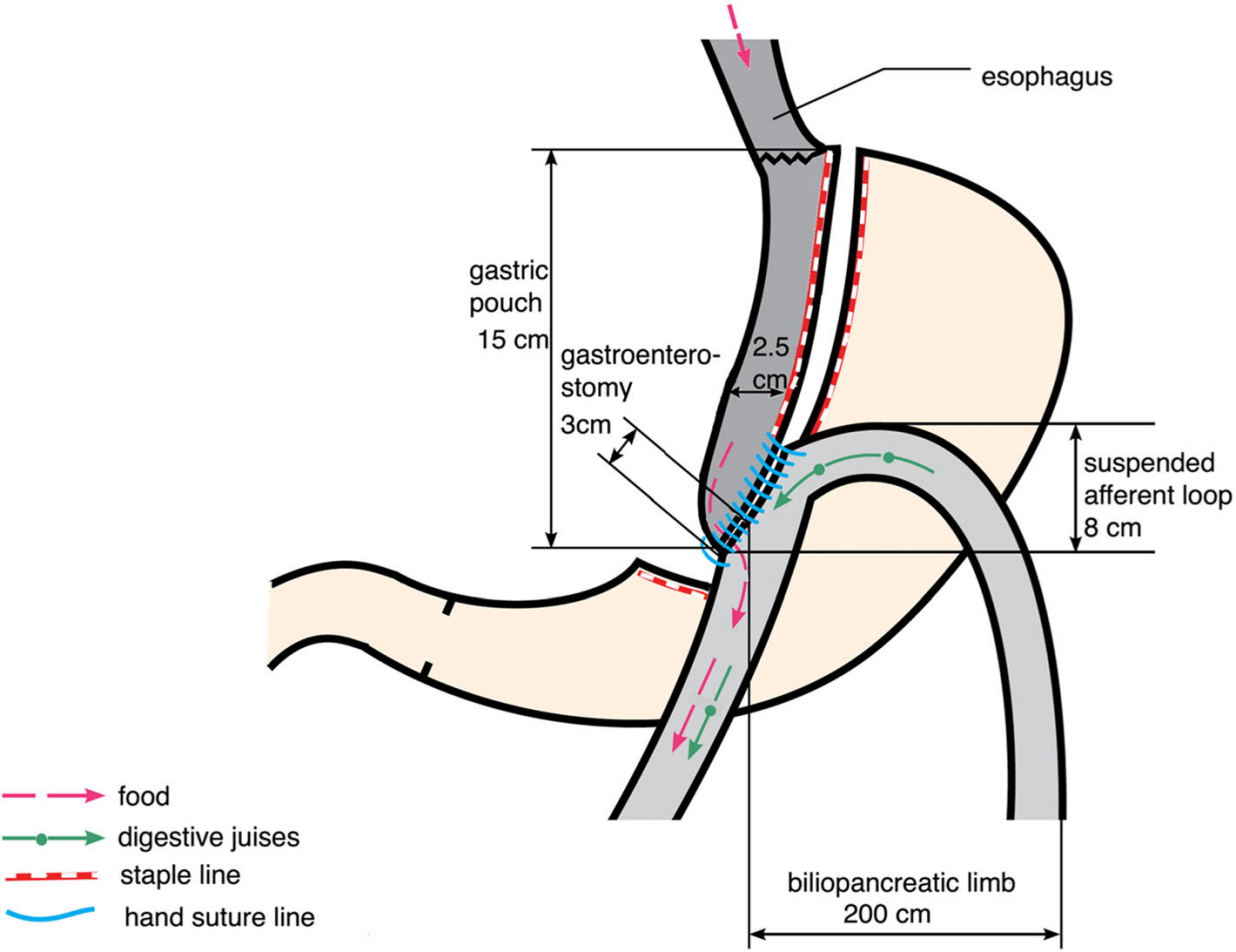
Laparoscopic one anastomosis (mini) gastric bypass was performed by Carbajo MA© 2021 Oral Ospanov

Experimental surgical bariatric procedure in the first (A) group: patients (*n*=50) underwent laparoscopic anastomosis (mini) gastric bypass with total wrapping of the fundus of the gastric excluded part and suture cruroplasty if hiatal hernia was present (FundoRingMGB group) (Fig. 1) [13]; active comparator surgical bariatric procedure in the second (B) group: patients (*n*=50) underwent laparoscopic anastomosis (mini) gastric bypass by Carbajo MA (Fig. 2) [14]. Only suture cruroplasty was performed if hiatal hernia was present (LOAGB group).

### Criteria for discontinuing or modifying allocated interventions {11b}

Not applicable

### Strategies to improve adherence to interventions {11c}

Not applicable

### Relevant concomitant care permitted or prohibited during the trial {11d}

Relevant concomitant care and interventions that are permitted during the trial: using drainage in the abdominal cavity for the first 4 days after surgery.

Relevant concomitant care and interventions are prohibited during the trial: balloon dilatation in the fundoplication area after surgery.

### Provisions for posttrial care {30}

Compensation for those who suffered from participation in the trial is not provided. Elimination of treatment gaps is carried out on a general basis in the national health system.

### Outcomes {12}

#### Primary outcome measure

1. Change of body mass index: The measure assesses a change of body mass index. Weight (kg) and height (cm) will be combined with the body mass index (BMI) kg/m^2^. Time frame: Baseline at 12, 24, and 36 months after surgery. Ideal body weight was calculated as 25 × [patient’s height in meters]^2^. Effectiveness endpoint follow-up included body mass index (BMI, kg/m^2^), change in BMI (ΔBMI [initial BMI − postoperative BMI]), excess BMI loss (EBMIL, % [(ΔBMI/initial BMI − 25) × 100]), and total weight loss (TWL, % [initial weight − postoperative weight/initial weight × 100]).

#### Secondary outcome measure

2. Postoperative bile reflux in the esophagus. The endoscopic finding of postoperative bile reflux in the esophagus. Mild bile reflux (BR) was assessed if the biliary reflux was asymptomatic and detected only by an endoscopic picture of gastritis near the gastroenteroanastomosis. A grade of average BR was assessed by the presence of pain or total gastritis of the pouch caused by the presence of bile. A grade of severe BR was assessed if BR symptoms of stomach pain or heartburn were intolerable or by endoscopic verification of esophagitis by the presence of bile in the esophagus [15]. Time frame: at 12, 24, and 36 months after surgery.
3. Assessment of GERD if preoperative GERD symptoms (GERD-HRQL) were present or if GERD symptoms occurred postoperatively (de novo GERD).

#### Participant timeline {13}

The flow diagram of the study design is shown in Table 1.

**Table 1:** The schedule of enrollment, interventions, and assessments of the FundoRingMGB trial

	Study period					
	Enrollment	Allocation	Post-allocation			Close-out
**Timepoint**	**V1 (preoperative)**	**V2 (operation)** ***March–December 2021***	**V3 12 months**	**V4 24 months**	**V5 36 months**	**April 2024**
**Enrolment:**						
**Eligibility screen**	X					
**Informed consent**	X					
**Allocation**		X				
**Interventions:**						
***[***FundoRingOAGB group***]***		X				
***[****L*OAGB group***]***		X				
**Assessments:**						
**Body mass index**	X	X	X	X	X	
**Change of body mass index**			X	X	X	
**Postoperative bile reflux in the esophagus**			X	X	X	
**GERD symptoms** or postoperative de novo GERD symptoms	X	X	X	X	X	
**Statistical analysis**						X

### Sample size {14}

Purpose sampling

- Effect size (*d*) (postoperative change in BMI (%)) = 15%.
- SD = 10.

Formula for calculating sample size is:

$$ n=2\ \left( Z\alpha +Z\left[1-\beta \right]\right)2\times \mathrm{SD}2/\mathrm{d}2 $$

Level of significance = 5%, power = 80% (Sample size increases as power increases. Higher the power, lower the chance of missing a real effect), *Zα* = *Z* is constant set by convention according to accepted α error and *Z* (1-*β*) = *Z* is constant set by convention according to the power of study [16].

Sample size calculations in our study:

$$ \mathrm{Z}\alpha =1.96,\mathrm{Z}\ \left(1-\beta \right)=1.28,\mathrm{SD}=10,\mathrm{d}\ \left(\mathrm{effect}\ \mathrm{size}\right)=15 $$

*n* = 2 (1.96 +   1.28) 2 × 102/152 = 9.33 individuals in each group should be recruited in the study. However, we plan to include more than five times the number of patients in the sample.

### Recruitment {15}

We will sample a homogenous population group (obese patients with body mass index (BMI) from 30 to 50 kg/m^2^), thus reducing the standard deviation and hence the sample size. We will recruit 100 obese patients (50 patients in two groups).

## Assignment of interventions: allocation

### Sequence generation {16a}

Informed consent will be obtained from each participant before patient enrollment in the study. Patients who meet all of the inclusion criteria and none of the exclusion criteria will be consecutively included and randomized into one of the two study arms by the study statistician, who is not involved in the enrollment, assignment, or assessment of patients, according to a random allocation sequence generated by Stata 7.0. The randomization list is kept strictly confidential. Allocation concealment is ensured by using sequentially numbered, identical, opaque, sealed envelopes (*n* = 100). Patients will be randomized to one of two groups at a 1:1 allocation ratio.

### Concealment mechanism {16b}

Sealed envelopes will be used during the visit before surgery.

### Implementation {16c}

The intervention will be communicated to the patient by a nurse who has no involvement in the enrollment or assessment of patients and who will open the sealed envelope during the visit before surgery.

## Assignment of interventions: blinding

### Who will be blinded {17a}

We will use an open-label trial, or open trial, when a type of clinical trial in which information is not withheld from trial participants. Bath, our open-label trial, is still randomized.

### Procedure for unblinding if needed {17b}

Not applicable.

## Data collection and management

### Plans for assessment and collection of outcomes {18a}

Treatment-related data were collected at V1 (baseline). According to the study protocol, follow-up data will be collected from V2 to 12 months, 24 months (V3), and 36 months after surgery (V4) (Table 1).

### Plans to promote participant retention and complete follow-up {18b}

Data collection begins on the day a participant signs the informed consent form and continues until the termination of the trial or until the participant withdraws from the trial for any reason. If participants discontinue or deviate from the study protocols, the investigators will attempt to minimize the missing data. All original data are kept in chronological order for verification.

### Data management {19}

Original data are transferred in a timely manner to a paper-based case report form (CRF) and an electronic database system located in a guarded facility at the trial site. Access to the study data is restricted. The PI will have access to the final dataset. An independent steering committee will monitor and examine adherence to the study protocol.

### Confidentiality {27}

The relevant regulations of the data protection legislation will be maintained.

The participants’ confidentiality was maintained at all times. For confidentiality reasons, case report forms (CRFs) do not contain any personal data on study participants. Members of the ethics committees are obliged to respect confidentiality and to refrain from divulging the participants’ identities or any other personal information they might be aware of. Source data in the hospital’s electronic patient information systems are secured by personal passwords and medical secrecy is maintained.

### Plans for collection, laboratory evaluation, and storage of biological specimens for genetic or molecular analysis in this trial/future use {33}

Not applicable.

## Statistical methods

### Statistical methods for primary and secondary outcomes {20a}

Statistical analysis will be performed using Microsoft Excel for Mac (Microsoft Corp., Redmond, WA) and StatPlus: MacPro (AnalystSoft Inc., Walnut, CA).

The normal distribution of the variables will be tested using the Kolmogorov–Smirnov test. Subsequently, quantitative demographic and outcome variables will be reported as the means and standard deviations (SD); qualitative variables will be reported as counts and percentages. Between-group comparisons among quantitative variables will be performed using independent one-way ANOVA. Where ANOVA omnibus F test is significant, pairwise comparisons using Fisher's least significant difference (LSD) method will be performed. Between-group differences among qualitative variables will be assessed using the chi-square test or Fisher's exact probability test. Measures of within-group change in quantitative variables, from baseline out to 12, 24, and 36 months, will be analyzed using the dependent-samples *t* test. Correlation and linear regression analyses will be performed to assess the strength and direction of the relationship between changes in BMI and telomere length and to estimate the predictive value of BMI change relative to telomere length at the 12-, 24-, and 36-month follow-ups. Statistical significance was set at *p* < 0.05.

### Interim analyses {21b}

The PI will have access to interim results and make the final decision to terminate the trial.

### Methods for additional analyses (e.g., subgroup analyses) {20b}

Not applicable

### Methods in analysis to handle protocol nonadherence and any statistical methods to handle missing data {20c}

Randomized clinical trials analyzed using the intent-to-treat (ITT) approach provide unbiased comparisons between treatment groups. In cases of nonadherence, intent-to-treat analysis will be performed to avoid overlap and exclusion effects that could disrupt the random assignment of treatment groups in the study. The ITT analysis will provide information on the potential effects of treatment policies rather than on the potential effects of a particular treatment.

### Plans to give access to the full protocol, participant-level data, and statistical code {31c}

Not applicable.

## Oversight and monitoring

### Composition of the coordinating center and trial steering committee {5d}

The principal investigator (PI) is responsible for the overall project and for organizing steering committee meetings. An independent steering committee will be responsible for ensuring the overall safety of participants, coordinating study meetings, supervising the study, monitoring data safety, and overseeing the quality control.

### Composition of the data monitoring committee, its role, and reporting structure {21a}

The composition of the data monitoring committee (DMC) is not needed because the PI organizes an independent steering committee meeting.

### Adverse event reporting and harms {22}

An adverse event (AE) refers to any untoward event that occurs during the clinical study but that does not necessarily have a causal relationship with the surgical treatment. Safety evaluations are performed from the point at which the signature on the informed consent form is obtained until the end of the study or until the patient withdraws from the trial, according to the management requirements. AEs and serious adverse events (SAEs) will be reported.

An SAE is an event that causes hospitalization, prolonged hospitalization, disability, incapacity, life-threatening illness or death, or congenital malformation during the clinical trial.

During the study, all AEs will be recorded. These records include the type of AE (using standard medical terminology), the date of the AE, the date of the disappearance/stabilization of the AE, the severity of the AE, the impact of the AE on the surgery, the relationship of the AE with the surgery, the treatment measures, and the outcomes. If an SAE occurs, researchers fill out an SAE report form. The report is signed and dated and reported to the Ethics Committee of The Society of Bariatric and Metabolic Surgeons of Kazakhstan (Nur-Sultan, Kazakhstan) within 24 h.

### Frequency and plans for auditing trial conduct {23}

Frequency for auditing trial conduct: 12, 24, and 36 months after surgery.

The process will be independent from investigators and the sponsor.

### Plans for communicating important protocol amendments to relevant parties (e.g., trial participants, ethical committees) {25}

If the acceptance criteria, the results, and analyses are changed, this information will be communicated to all trial participants and to the ethics committee in writing (letters).

A sponsor will submit a protocol amendment in cases when there are changes in the existing protocol that significantly affect the safety of subjects, scope of the investigation, or scientific quality of the study.

### Dissemination plans {31a}

The trial results will be distributed in journal publications.

## Discussion

Advances in bariatric and metabolic surgery make it possible to include this subspecialty in our national health systems as an independent specialization [17]. LOAGB is recognized as a simple and safe gastric bypass method [12], and LOAGB has been shown to match RYGB in weight loss and metabolic improvement after 2 years [18].

However, the high likelihood of intractable biliary reflux in LOAGB, 2.8% after revisional bariatric procedures (r-LOAGB) and 0.4% after primary LOAGB (p-LOAGB), is a serious complication. A. Soprani et al. [19] collected a series of 16 patients who underwent laparoscopic Nissen/MGB for large sliding hiatal hernia or paraesophageal hernia between 2013 and 2016. The surgery consisted of a standard MGB combined with crural repair and Nissen fundoplication using the remnant stomach as an anti-reflux valve. During this period, ten patients underwent Nissen/MGB after laparoscopic adjustable gastric band (LAGB) (seven two-stage and three one-stage procedures). None of these patients developed postoperative symptomatic bile reflux.

Our current study, for the first time, investigates the results of applying a similar method for all cases of morbid obesity according to the specified inclusion criteria. Thus, regardless of the presence or absence of insufficiency of the lower esophageal sphincter and hiatal hernia, in the experimental group, we will perform a bariatric surgical procedure, which we called “FundoRingOAGB.” The combination of gastric bypass and fundoplication (CGB&F) as described in the publication can be classified into three main types:

- Type 1 CGB&F—One-stage: simultaneous gastric bypass and fundoplication.
- Type 2 CGB&F—Two-stage: consequent gastric bypass after fundoplication with (a) complete takedown of the wrap; (b) partial takedown of the wrap; and (c) without takedown of the wrap.
- Type 3 CGB&F—Two-stage: sequential fundoplication after a previous bariatric surgery.
- Type 1 CGB&F includes primary bariatric procedures, and types 2–3 are secondary surgical procedures [13].

The return of weight after any gastric bypass method significantly reduces the preference for using bariatric and metabolic surgery for the treatment of morbid obesity; therefore, we first examined the likelihood of weight return after the experimental (FundoRingOAGB) and standard procedure of LOAGB. For the primary outcome measure, the change in body mass index will be assessed 12, 24, and 36 months after the surgical procedure.

Simultaneously, secondary outcome measures were the likelihood of biliary reflux within 12, 24, and 36 months after this procedure. Finally, GERD will be assessed if preoperative GERD symptoms are present or if GERD symptoms occur postoperatively (de novo GERD). For this aim, we will use a quality-of-life scale for gastroesophageal reflux disease (GERD-HRQL) [20].

In the present randomized controlled trial, we expect to show that one anastomosis gastric bypass with fundoplication ring for obese patients will better prevent the return of weight and bile reflux than the standard method. The idea to investigate in a randomized study the combination of two procedures as a primary procedure for one-anastomotic gastric bypass was expressed by Oral Ospanov [13, 21].

## Trial status

At the time of the initial manuscript submission, recruitment had started on March 29, 2021, and the last patient is expected to be included in the study in December 2021. The study will be completed on April 5, 2024.

## Abbreviations

AE: Adverse event
ASA: American Society of Anesthesiologists
BMI: Body mass index
BR: Bile reflux
CRF: Case report form
DMC: Data monitoring committee
EBMIL: Excess body mass index loss
GERD: Gastroesophageal reflux disease
ITT: Intent to treat
LMGB: Laparoscopic minigastric bypass gastric bypass
LOAGB: Laparoscopic one anastomosis gastric bypass
LRYGB: Laparoscopic Roux-en-Y gastric bypass
LSD: Least significant difference
PI: Principal investigator
REC/IRB: Research ethics committee/institutional review board
SAEs: Serious adverse events
SBMSK: Society of Bariatric and Metabolic Surgeons of Kazakhstan
SD: Standard deviation
TWL: Total weight loss
